# Genotype-First Approach Identifies an Association between rs28374544/FOG2^S657G^ and Liver Disease through Alterations in mTORC1 Signaling

**DOI:** 10.3390/genes15081098

**Published:** 2024-08-21

**Authors:** Donna M. Conlon, Siri Kanakala, Tess Cherlin, Yi-An Ko, Cecilia Vitali, Sharavana Gurunathan, Rasika Venkatesh, Jakob Woerner, Lindsay A. Guare, Penn Medicine Biobank, Anurag Verma, Shefali S. Verma, Marie A. Guerraty

**Affiliations:** 1 Department of Medicine, University of Pennsylvania Perelman School of Medicine, 3400 Civic Center Blvd., Philadelphia, PA 19104, USA; dconlon@pennmedicine.upenn.edu (D.M.C.); anurag.verma@pennmedicine.upenn.edu (A.V.); 2 Department of Pathology and Laboratory Medicine, University of Pennsylvania Perelman School of Medicine, Philadelphia, PA 19104, USAshefali.setiaverma@pennmedicine.upenn.edu (S.S.V.); 3 Department of Genetics, University of Pennsylvania Perelman School of Medicine, Philadelphia, PA 19104, USArasika.venkatesh@pennmedicine.upenn.edu (R.V.); 4 Department of Biostatistics, Epidemiology, and Informatics, University of Pennsylvania Perelman School of Medicine, Philadelphia, PA 19104, USA; jakob.woerner@pennmedicine.upenn.edu; 5 Perelman School of Medicine, University of Pennsylvania, Philadelphia, PA 19104, USA

**Keywords:** FOG2, mTORC1, MAFLD, Functional Genomics

## Abstract

Metabolic dysfunction-associated Fatty Liver Disease (MAFLD) has emerged as one of the leading cardiometabolic diseases. Friend of GATA2 (FOG2) is a transcriptional co-regulator that has been shown to regulate hepatic lipid metabolism and accumulation. Using meta-analysis from several different biobank datasets, we identified a coding variant of FOG2 (rs28374544, A1969G, S657G) predominantly found in individuals of African ancestry (minor allele frequency~20%), which is associated with liver failure/cirrhosis phenotype and liver injury. To gain insight into potential pathways associated with this variant, we interrogated a previously published genomics dataset of 38 human induced pluripotent stem cell (iPSCs) lines differentiated into hepatocytes (iHeps). Using Differential Gene Expression Analysis and Gene Set Enrichment Analysis, we identified the mTORC1 pathway as differentially regulated between iHeps from individuals with and without the variant. Transient lipid-based transfections were performed on the human hepatoma cell line (Huh7) using wild-type FOG2 and FOG2^S657G^ and demonstrated that FOG2S^657G^ increased mTORC1 signaling, de novo lipogenesis, and cellular triglyceride synthesis and mass. In addition, we observed a significant downregulation of oxidative phosphorylation in FOG2^S657G^ cells in fatty acid-loaded cells but not untreated cells, suggesting that FOG2^S657G^ may also reduce fatty acid to promote lipid accumulation. Taken together, our multi-pronged approach suggests a model whereby the FOG2^S657G^ may promote MAFLD through mTORC1 activation, increased de novo lipogenesis, and lipid accumulation. Our results provide insights into the molecular mechanisms by which FOG2^S657G^ may affect the complex molecular landscape underlying MAFLD.

## 1. Introduction

Metabolic dysfunction-associated fatty liver disease (MAFLD) is a leading cardiometabolic disease characterized by dysregulation of hepatic lipid metabolism and accumulation of triglyceride (TG) in the liver. If left untreated, it can progress from steatosis to metabolic dysfunction-associated steatohepatitis (MASH)**,** cirrhosis, and, increasingly, hepatocellular carcinoma (HCC). Both globally and in the United States, MAFLD is causing increased disease burden and mortality and will soon be the leading cause of liver transplantation [1].

Friend of GATA 2 (FOG2) is a transcriptional co-regulator encoded by the *ZFPM2* gene that is crucial for both development and organ homeostasis in adulthood [2,3]. We have recently identified a coding variant of *FOG2* (rs28374544, A1969G, Ser657Gly, hereafter referred to as FOG2^S657G^), which is highly present in individuals of African ancestry (MAF~0.20) and associated with coronary microvascular disease [4]. In addition to cardiac expression, FOG2 is also present in the developing and adult liver. It has been shown to affect insulin resistance and hepatic fat accumulation through the regulation of *Ppara* in the liver and to regulate AKT [5]. Single nucleotide RNAseq of whole livers in humans found that FOG2 expression was increased in hepatocytes from livers of individuals with MASH as compared to those with MAFLD [6]. Higher levels of FOG2 expression were also associated with a more favorable prognosis in a study of hepatocellular carcinoma in humans [7].

Based on these data, we hypothesized that FOG2^S657G^ may play a role in liver homeostasis. Here, we report human genetic evidence linking FOG2^S657G^ with liver disease and injury and hepatic lipid metabolism. We used gene expression data from iPSCs-differentiated to hepatocytes to identify pathways through which FOG2^S657G^ may regulate hepatocyte biology. We show that FOG2^S657G^ increases mTORC1 signaling and, in turn, promotes lipid accumulation and affects cellular metabolism.

## 2. Results

### 2.1. FOG2^S657G^ Is Associated with Liver Injury and Disease in Multiple Biobank Populations

In order to determine whether FOGS657G was associated with human liver phenotypes, we first used Genebass, which encompasses 4529 phenotypes with gene-based and single-variant testing across 394,841 individuals with exome sequence data from the UK Biobank (UKBB) (https://app.genebass.org accessed on 29 March 2024) [8].There was an association between rs28374544 and liver failure/cirrhosis phenotype (*p* = 0.0053375) in this population. Given the relatively low representation of African ancestry individuals in the UKBB and the prevalence of *FOG2^S657G^* in individuals of African ancestry, it is possible that the signal that we observed was due to ancestry and not the variant. Therefore, we performed a meta-analysis for rs28374544 in the PennMedicine Biobank (PMBB) [9], All of Us Biobank [10], and the Million Veterans Program (MVP) [11]. Datasets include only individuals of African ancestry [12]. Here, we observed a significant increase in “cirrhosis of the liver without mention of alcohol” (*p* = 0.01239, β = +0.068) (Table 1).

We next examined the levels of serum proteins in 389 PMBB participants of African ancestry who had undergone genotyping and proteomic profiling with SOMAscan (SomaLogic) for markers of liver disease and liver injury [13]. The mean age of participants was 61 ± 13.6, and 58% were male. We found that there was a 30% increase in plasma alanine transaminase (ALT) with at least one copy of FOG2^S657G^ (Table 1). Taken together, these data support a positive association between FOG2^S657G^ and liver disease.

### 2.2. Genomic Analysis from a Cohort of Induced Pluripotent Stem Cell Lines Differentiated into Hepatocytes Idenfies an Association between FOG2^S657G^ and mTORC1 Pathway

**Figure 1:**
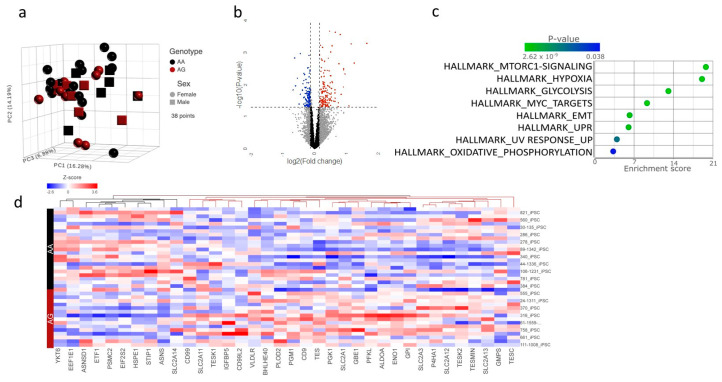
RNAseq analysis of iHeps from individuals with and without FOG2^S657G^ identifies mTORC1 signaling as the top differentially regulated pathway. (**a**) Principal component analysis of the normalized RNAseq data in AA (black) and AG (crimson) iHeps derived from both male (round) and female (square) individuals of African American Ancestry in Penn Medicine Biobank. (**b**) Volcano plot of differentially expressed genes as determined by DeSeq2 analysis. Blue dots indicate significantly downregulated and red dots significantly upregulated (nom *p* < 0.05 and log2 (Fold change) > 1.2). (**c**) Gene set enrichment analysis of differentially expressed genes using Hallmark pathways. (**d**) Heat map of differentially expressed genes in the mTORC1 hallmark pathway consistent with an increase in mTORC1 signaling in individuals with the variant.

To gain insight into mechanisms by which FOG2^S657G^ may alter hepatocyte gene expression, we examined the effects of rs28374544 on gene expression in a cohort of human induced pluripotent stem cell (iPSC) lines differentiated into hepatocytes (iHeps) previously reported [14]. This dataset includes RNAseq data from 38 iHeps lines isolated from self-reported African American individuals (25 females, 13 males), 23 of which carry the reference allele (AA) and 15 of which are heterozygous for the variant (AG) (Figure 1a). We performed differential gene expression analysis and found that 152 genes were upregulated and 131 were downregulated in lines from iHeps with FOG2 ^S657G^ at a nominal *p*-value of 0.5 and log2 fold change of 1.2 (Figure 1b). The top differentially expressed gene was *IGFBP2* (fold change = 1.38, *p* = 2.1 × 10^−4^), which encodes the Insulin growth factor binding protein 2 (IGFBP2) and has been reported to be associated with MAFLD and progression to MASH [15,16] (Table S1) Other top differentially expressed and abundant genes included ENO1, ALDOA, PKM, and PGK1, key regulators of various metabolic pathways, including notably anaerobic respiration, hypoxia, glycolysis, and oxidative phosphorylation (Table S1).

We then performed Gene Set Enrichment Analysis (GSEA) using the differentially expressed genes and Hallmark gene sets, which represent canonical pathways. The most differentially regulated pathway was mTORC1 signaling (Enrichment score 19.8, *p* = 2.6 × 10^−9^, Figure 1c). Related pathways, including hypoxia, glycolysis, and oxidative phosphorylation, were also differentially regulated. Closer examination of the genes differentially regulated in the mTORC1 pathway was consistent with an increase in mTORC1 signaling (Figure 1d), which has been shown to affect hepatic lipid metabolism in several ways, including driving de novo lipogenesis (DNL).

### 2.3. Functional In Vitro Studies of FOG2^S657G^ Overexpression in Huh7 Cells Confirm Regulation of mTORC1, DNL, and TG Synthesis

**Figure 2:**
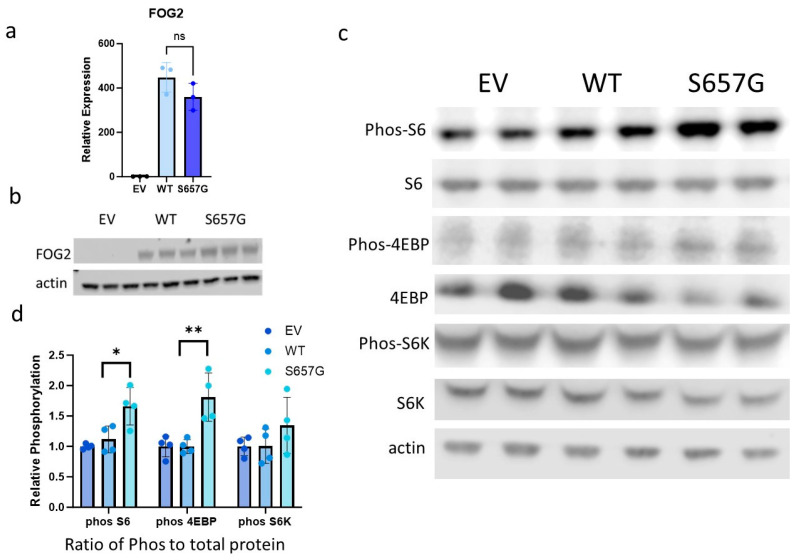
FOG2^S657G^ increases mTORC1 signaling in Huh7 cells. Huh7 cells were transiently transfected with vectors expressing control empty vector (EV), FOG2 (WT), or FOG2^S657G^ (S657G). (**a**,**b**) Gene and protein expression measured 72 h post-transfection, using qRT-PCR and Western blot, respectively, confirms increased and similar expression of FOG2 WT and S657G relative to EV. ns = not significant (**c**) Representative Western Blot of phosphorylated and total fractions of mTORC1 targets in transfected Huh7 cells. (**d**) Quantification of the ratio of phosphorylated to total protein using Image J analysis, n = 4/group * *p* < 0.05, ** *p* < 0.01 by Student’s *t*-test.

To gain mechanistic insights into the associations identified via genomic analyses, we turned to in vitro functional studies. To determine the role of FOG2^S657G^ on mTORC1 signaling, we transfected Huh7 cells with vectors expressing empty vector, wild type (WT) FOG2, or FOG2^S657G^ under EF1alpha promoter. These resulted in similar levels of gene and protein over-expression (Figure 2a,b). Gene expression of *IGFBP2* was significantly increased (1.9-fold, *p* < 0.001) in cells overexpressing FOG2^S657G^, similar in direction and magnitude to what was observed in FOG2^S657G^ expressing iHeps (Figure S1). Using western blot analysis of whole cell lysates, we found that there was significantly higher phosphorylation of S6 and 4EBP in FOG2^S657G^-expressing cells as compared to WT (Figure 2c,d). Increased phosphorylation of these mTORC1 targets is consistent with increased mTORC1 signaling in the cells and increased changes in metabolic pathways that are important for cell survival and proliferation and are often altered in disease states [17].

**Figure 3:**
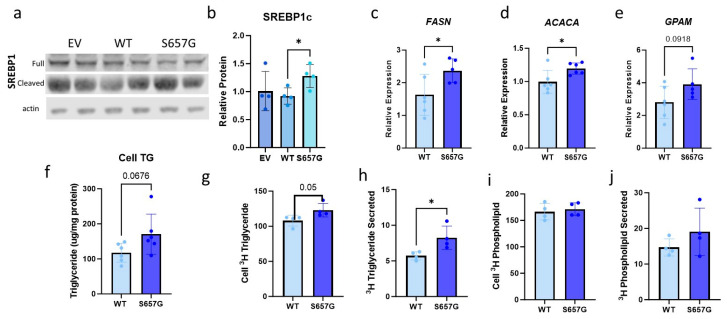
FOG2^S657G^ expression in Huh7 cells results in increased de novo lipogenesis and TG synthesis. (**a**) Representative Western blot of total cell lysate for SREBP1c levels. (**b**) Quantification of the amount of cleaved SREBP1c as normalized to actin levels using ImageJ, n = 4/group. (**c**–**e**) RNA expression of DNL genes in WT and S657G transfected Huh7 cells show significantly increased expression of key regulators of DNL. n = 6/group (**f**) Total cell TG mass was normalized to total protein as measured by BCA and expressed as triglyceride per mg protein.n = 6/group (**g**–**j**) Transfected cells were labeled overnight with 3H glycerol, and the total amount of 3H TG in the cell (**g**) and the media (**h**) and 3Hphospholipid in cell (**i**) and media (**j**) was measured and normalized to total cell protein. n = 4/group, * *p* < 0.05 by Student’s *t*-test.

In addition to regulating protein and nucleotide translation, nutrient sensing, cell growth, and autophagy, mTORC1 is also known to promote the cleavage and nuclear localization of SREBP1c to drive the transcription of genes related to DNL [18]. To determine if the increase in mTORC1 signaling affected lipid metabolism, we measured SREBP1 protein and observed a 35% increase (*p* < 0.05) in the active cleaved form of SREBP1 in cells overexpressing FOG2^S657G^ relative to WT with no difference in total SREBP1 protein (Figure 3a,b). Since the nuclear form of SREBP1 can drive the transcription of genes related to DNL, we measured the relative gene expression of key genes involved in DNL, including *FASN*, *ACACA*, and *GPAM* (Figure 3c–e). This was associated with an increase in cell TG mass (Figure 3f). To determine whether this was due to an increase in TG synthesis in the cells, we labeled cells with ^3^H glycerol and measured the incorporation of ^3^H glycerol into both TG and phospholipid (PL). There was increased intracellular ^3^H-TG in FOG2^S657G^-expressing cells, indicating an increase in TG synthesis (Figure 3g). There was also increased ^3^H-TG in the media which is consistent with an increase in TG secretion due to more cellular TG availability (Figure 3h). There was no difference in cellular or secreted ^3^H-PL, demonstrating that there is a specific effect on TG synthesis and not a result of a difference in label uptake (Figure 3i,j).

### 2.4. FOG2^S657G^ Cells Have Reduced Oxidative Phosphorylation in iHeps and Huh7 Cells

**Figure 4:**
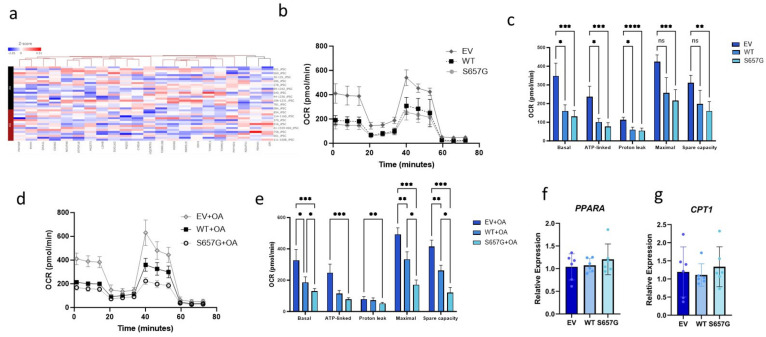
Decreased oxidative phosphorylation with FOG2^S657G^ expression. (**a**) Heat map of differentially expressed genes in the iHeps in the Hallmark oxidative phosphorylation pathway is consistent with decreased activity in the pathway. (**b**–**e**) Seahorse Bioanalyzer data in Huh7 cells transfected with FOG2 or FOG2^S657G^. Oxygen consumption rate (OCR) is measured in cells over 75 min in non-treated cells (**b**,**c**) other transfected cells were incubated with 0.2 mM OA overnight (**d**,**e**) prior to measurement. All conditions represent 10–12 wells * *p* < 0.05, ** *p* < 0.01, *** *p* < 0.001, **** *p* < 0.0001, ns = not significant (**f**,**g**) RNA expression of PPARA and CPT1 genes in WT and S657G transfected Huh7 cells.

Another differentially regulated pathway in iHep lines from individuals with the FOG2^S657G^ variant was oxidative phosphorylation (Figure 1c), and the directionality of the differentially expressed genes of this pathway was suggestive of decreased oxidative phosphorylation (Figure 4a). A reduction in oxidative phosphorylation can also contribute to lipid accumulation. We, therefore, measured the oxygen consumption rate (OCR) in transfected cells using the Seahorse bioanalyzer. We found that both FOG2 and FOG2^S657G^ transfected cells had lower OCR as compared to EV control cells under basal conditions, and there was no significant difference between FOG2^S657G^-expressing cells and WT control (Figure 4b,c). However, when cells were lipid-loaded with 0.2 mM oleic acid (OA) for 16 h prior to the assay, we noted a decrease in both basal and maximal respiration in FOG2^S657G^-expressing cells relative to control (Figure 4d,e). This decrease in oxidative phosphorylation in the presence of fatty acid loading suggests that there may be a decrease in fatty acid oxidation, which may contribute to the lipid accumulation phenotype observed in the cells. Prior studies have shown that Fog2 may regulate *Ppara* expression and affect fatty acid oxidation in the mouse liver. However, we found no difference in the expression of *Ppara* or its target CPT1 (Figure 4f,g).

## 3. Discussion

We have identified an association between FOG2^S657G^ and liver disease/injury and increased plasma ALTs. Genomic analysis from multiple human iPSC lines differentiated to iHeps suggested an association between FOG2^S657G^ and metabolic pathways, including mTORC1 signaling, hypoxia, glycolysis, and oxidative phosphorylation. In vitro functional studies showed that FOG2^S657G^ can activate hepatocyte mTORC1 to increase DNL, increase TG synthesis, and decrease oxidative phosphorylation. Together, these act to increase hepatic TG accumulation. 

In addition to regulating hepatic lipid metabolism [5], FOG2 has also been shown to affect metabolic pathways in other cell types. A mutation in Fog2’s LXCX retinoblastoma protein binding motif has been shown to regulate metabolic processes such as adipogenesis, weight gain, and small intestine homeostasis in mice [19,20]. Guo et al. demonstrated that overexpression of Fog2 in the liver protected mice from the development of fatty liver but, interestingly, was associated with an increase in insulin resistance [5]. The reciprocal results of increased fatty liver and protection from insulin resistance were observed with Fog2 siRNA knockdown experiments and these effects were mediated through altered expression of *Ppara* in the liver. Interestingly, this group also observed that Fog2 could affect the phosphorylation of AKT (an upstream regulator of mTORC1) and thus alter the insulin signaling pathway [5]. This is consistent with previous results that showed that the drosophila homolog of Fog2, U-Shaped, and human FOG2 inhibits phosphoinositide-3 kinase (PI3K) and results in decreased phosphorylation of AKT and reduced cell growth in drosophila and human Hep3B cells [21]. Additionally, they showed that the middle region of FOG2 from amino acid 507–789 is the domain that binds directly to the P85 subunit of PI3K and inhibits its activity. The FOG2^S657G^ mutation is in this region and suggests that the effect of the amino acid substitution might be to alter the interaction of FOG2 with PI3K in the cytosol. If FOG2^S657G^ prevented the interaction with PI3K, then this might result in increased activity, increased AKT phosphorylation, and ultimately increased activation of the downstream mTORC1 pathways.

Based on the results of Guo et al., FOG2^S657G^-based lipid accumulation would result in a loss of function. FOG2^S657G^ has previously been reported to serve as loss-of-function in failing to repress GATA4 in the heart [22]. However, activation of the mTORC1 pathway in FOG2^S657G^ hepatocytes is more consistent with the development of insulin resistance, which was seen with the FOG2 overexpression [5]. These discrepant results suggest more complex interactions of FOG2 in hepatocytes. For example, FOG2^S657G^ could mediate changes via transcriptional regulation in the nucleus while at the same time exerting different effects in the cytoplasm through regulation of AKT and downstream pathways. Therefore, it is possible that hepatocyte FOG2^S657G^ results in a change of function rather than a simple loss or gain of function in regard to hepatocyte metabolism.

Prior human GWAS studies for hepatic steatosis have not identified this variant [23,24].

One potential reason is that FOG2^S657G^ is primarily present in individuals of African ancestry. There are known disparities in the diagnosis of MAFLD, with lower reported incidence of MAFLD in African American relative to Hispanic and Caucasian individuals [25,26]. African American individuals have also been traditionally underrepresented in population-based studies of MAFLD [27]. Most MAFLD studies are relatively small due to the diagnostic difficulty and include an even smaller number of patients of African ancestry, limiting the ability to detect this specific variant. For example, only 1–2% of individuals in the UK Biobank have African ancestry, limiting the discovery of relevant new variants in this population [28]. More recent studies in a larger multi-ancestry cohort also did not identify this variant [29]. Additionally, analyses using biobank data are subject to participation bias, particularly when individuals are from a healthcare population, such as PMBB or MVP. These populations may have a higher incidence of risk factors for liver disease, which can confound the signal for liver disease. However, we performed a meta-analysis using three different biobanks to help mediate the effects of bias. Furthermore, our in vitro studies demonstrate that there is biological plausibility for this variant altering hepatic lipid metabolism and contributing to liver disease.

Our analysis identified an association between liver disease phenotypes and ALT, which are more consistent with MASH. Our data from human biobanks and cell lines suggest mild TG accumulation with FOG2^S657G^. This phenotype is subtle, consistent with a point mutation resulting in a single amino acid change that is predicted to generate a slight conformational change [4]. It is clear that this signal is not driven by steatosis alone. This may be because the variant is exerting only minor effects on lipid metabolism and steatosis, or alternately, steatosis may not be driving liver injury. FOG2^S657G^ may be driving metabolic changes in other tissues and cell types that may contribute to the development of insulin resistance in the liver. For example, our analysis identified IGFBP2 as differentially regulated by FOG2^S657G^ in both iHeps and Huh7 cells. IGFBP2 has recently been identified to be the second-most differentially regulated protein in the progression from MAFLD to cirrhosis in an Icelandic population [30]. The mechanism by which IGFBP2 may affect this progression is not known; however, recent data suggest that zone 2 hepatocytes that express IGFBP2 play key roles in liver regeneration [31]. Additional studies are needed to understand the interactions between FOG2^S657G^, IGFBP2, and mechanisms of progression from MAFLD to cirrhosis.

Future studies in pre-clinical models of FOG2^S657G^ are needed to understand the mechanisms by which FOG2^S5657G^ drives hepatocyte lipid accumulation and may promote progression to liver injury. This would allow for the examination of other liver cell types and extra-hepatic tissues and their effect on liver injury. Currently, there are limited treatment options for MAFLD and MASH, and more investigation into disease pathogenesis is needed [32]. Understanding the mechanisms by which steatosis can develop, and progress is critical to developing new treatments. We have shown that FOG2^S657G^ is associated with mild TG accumulation and downstream sequelae such as elevated ALT and liver disease phenotypes. Elucidating the role of FOG2 and FOG2^S657G^, specifically in the liver, may reveal new pathways to target and novel treatment avenues.

## 4. Methods

### 4.1. Meta-Analysis of Biobanks

PheWAS for rs28354744 was performed in PMBB, All of Us, and MVP in individuals of African ancestry using SAIGE [12]. Each analysis included 10,995, 50,457, and 120,039 participants and 959, 1472, 945 phecodes, respectively. Fixed-effect meta-analysis was performed using the metagen function in the R package meta (version 6.5-0) [33]. Results from the PheWAS meta-analysis were combined with their respective phecodes using the R package PheWAS (version 0.99.5-4).

### 4.2. Plasma Protein Measurements

Plasma was collected from 389 African American individuals enrolled in the PMBB and stored at −80 °C before shipping on dry ice to SomaLogic, Inc. (Boulder, CO, USA) for measurement of the relative concentration of 1305 proteins using the multiplexed, aptamer-based SOMAscan assay, as previously described [13]. Data were normalized and log-transformed, and plasma ALT levels in the 102 carriers of rs28354744 were compared to non-carriers using linear regression analysis.

### 4.3. Analysis of Published iPSC-Hepatocyte Data

Raw count data were downloaded from GEO and annotated using biomaRt and EnsDb.Hsapiens.v86 packages in R 4.2.2. Partek Flow (11.0.23.0918) was used for all genomic analyses, including principal component, differential gene expression, pathway, and clustering analyses. DEseq2 was used to normalize data, generate normalized counts, and perform differential gene expression analysis. Gene Set Enrichment Analysis was performed for genes with a nominal *p*-value < 0.05 and log2(Fold change) > 1.2, using Hallmark pathways which represent canonical pathways. We report GSEA enrichment scores and all pathways with nominal *p* value of less than 0.05. Heatmaps represent hierarchical clustering using average linkage Euclidean distance.

### 4.4. Huh7 Cell Culture and Transfection

HuH-7 cells were obtained from the JCRB cell bank and maintained in DMEM with 10% fetal bovine serum (FBS) and 1% antibiotics. Prior to the assay, cells were plated collagen coated 6-well plates. Cells were transfected with empty vector (EV), WT FOG2, or FOG2^S657G^ (Twist Biosciences, San Francisco, CA, USA) using commercially available reagents (Lipofectamine 2000, OptiMEM, Invitrogen Carlsbad, CA, USA) according to manufacturer instructions. Experiments were performed 72 h after transfection.

### 4.5. Gene Expression

Gene expression was measured by quantitative real-time PCR (qRT-PCR). In brief, RNA was extracted from cell samples with a Qiagen RNA Easy Protect Kit, and 1 µg of RNA was reversed transcribed to cDNA using a High-Capacity cDNA Reverse Transcription Kit (Thermo, Carlsbad, CA, USA). The cDNA was diluted 30 times and used for qRT-PCR analysis using PowerUp SYBR Green Master Mix (Applied Biosystems, Carlsbad, CA, USA) and indicated primers. All samples analyzed were normalized to the average of 3 housekeeping using the delta Ct method and normalized to the empty vector control sample.
PrimerSequenceFOG2 FTGCTGGACTATCACGAGTGCFOG2 RGACATCAGGGCTGTTTCGTTFASN FCTTCCGAGATTCCATCCTACGCFASN RTGGCAGTCAGGCTCACAAACGACACA FGGAGAGCATGTCCAATGTTCCACACA RCGTCCTGTTCATTTCGTGCAAGPAM FTCTTTGGGTTTGCGGAATGTTGPAM RATGCACATCTCGCTCTTGAATAAB2M FGAGGCTATCCAGCGTACTCCAB2M RCGGCAGGCATACTCATCTTTTUBC FGTGGTGCGTCCAGAGAGACUBC RGGCCTTCGCCATATCCTTTTCIPO8 FTCCGAACTATTATCGACAGGACCIPO8 RGTTCAAAGAGCCGAGCTACAA

### 4.6. Western Blot

Cells were lysed in 1 mL of lysis buffer containing 62.5 mM sucrose, 0.5% sodium deoxycholate, 0.5% Triton X-100, 50 mM Tris-HCl, pH 7.4, 150 mM NaCl, 50 µg/mL leupeptin, 50 µg/mL pepstatin A and 30 µL/mL of a protease inhibitor. Protein concentration was measured by BCA protein reagent. Samples were subject to electrophoresis on 4–12% Bis-Tris gels (Thermo Fisher, Carlsbad, CA, USA) and transferred to nitrocellulose membranes. Membranes were incubated with the following antibodies at manufacturer-recommended dilutions: 

Fog2 (Santa Cruz, Dallas, TX, USA Cat# sc10755)

actin (Sigma, St Louis., MO, USA Cat#A5441) 

Phos-4E-BP1 (Cell Signaling Technology, Danvers, MA, USA Cat#9451)

Phos-S6 (Cell Signaling Technology, Danvers, MA, USA Cat# 5364)

Phos-S6K (Cell Signaling Technology, Danvers, MA, USA Cat#9234)

4E-BP1 (Cell Signaling Technology, Danvers, MA, USA Cat#9644)

S6 (Cell Signaling Technology, Danvers, MA, USA Cat#2217)

S6K (Cell Signaling Technology, Danvers, MA, USA Cat#2708) 

SREBP1 (Abcam, Cambridge, UK Cat#3259)

All primary antibodies were incubated overnight at 4 °C. Mouse or rabbit IRdye secondary IgG antibodies (LI-COR) (1:2500) were incubated for 1 h at room temperature. Protein bands were visualized using the LICOR Odyssey Fc imager (LI-COR). Bands were quantified using Image J software. Phospho-S6, Phospho S6K, and Phospho-4EBP were normalized to their respective total proteins. SREBP1c was normalized to actin.

### 4.7. TG Mass Assay

For detection of TGs by colorimetric enzymatic assay, HuH-7 cells were washed with PBS and incubated with 3:2 Hexane: Isopropanol for 2 h. The extracted lipid was dried under N_2_ and reconstituted in 300 μL of 15% Triton X-100 in chloroform and dried under N_2_ and reconstituted in 300 μL of water. Then, 20 μL of extracted lipid was used for colorimetric TG measurement by enzymatic assay (Thermo Fisher, Carlsbad, CA, USA) using commercial lipid standard. Total protein was recovered by solubilizing the cells in 0.1 N NaOH and measured using bicinchoninic acid (BCA) assay (Pierce, Thermo Fisher Scientific, Carlsbad, CA, USA).

### 4.8. TG Synthesis Assay

For detection of newly synthesized TGs, HuH-7 cells were incubated with 10 µCi/mL of 1,2,3-[3H] Glycerol (American Radiolabeled Chemicals, St Louis. MO USA) with 0.1 mM oleic acid (OA) for 4 h. Cell lipids were extracted from cells using 3:2 hexane: isopropanol mixture. Media lipids were extracted using a 2:1 chloroform: methanol mixture, according to the Folch method [34]. Extracts were dried under N2 gas and resuspended in hexane, spotted, and separated on silica 60 TLC plates. Lipid species were visualized using iodine vapor. Measured lipid counts were then normalized to the total cell protein from respective wells as described above.

### 4.9. Seahorse Bioanalyzer Assay

Transfected Huh7 cells were plated at a density of 20,000 cells/well 48 h after transfection. Some cells were treated with 0.2 mM OA overnight. Following incubation, cells were challenged with a Mito Stress Kit (2 μM Oligomycin, 1.5 μM FCCP, and 0.5 μM Rotenone/Antimycin A), and the oxygen consumption rate was measured using a Seahorse XFe96 Analyzer (Agilent, Wilmington, DE, USA). Oxygen consumption rate was normalized to the cell number using 2 μM Hoechst 33342 staining on BioTek Cytation 5 Cell Imaging Multimode Reader (Agilent), and key parameters like basal, maximal, and ATP-linked mitochondrial respiration were quantified.

### 4.10. Statistical Analysis

Statistical analysis was performed using Prism version 9.3.1 (350). Details of experiments are described in the figure legends, and data are presented as mean ± standard deviation unless otherwise stated. Differences in the mean values between the 2 groups were assessed using a 2-tailed Student’s *t*-test. Differences in mean values among more than 2 groups were assessed by 1-way ANOVA with any individual group differences determined using Tukey’s test. *p* < 0.05 was considered to be statistically significant.

## Figures and Tables

**Table 1:** Summary of liver-associated phenotypes associated with rs28374544.

Source	Measurement	β	*p*-Value	Case (N)	Total (N)
Genebass	Liver Failure/Cirrhosis	+4.288	0.0053	234	367,963
Meta-analysis: PennMedicine Biobank, All of Us, Million Veterans Program	Cirrhosis of the liver without mention of alcohol	+0.068	0.01239	5665	171,129
PennMedicine Biobank	Plasma alanine transaminase	+0.23	0.03	102	389

## Data Availability

The original contributions presented in the study are included in the article/Supplementary Material, further inquiries can be directed to the corresponding author/s.

## Supplementary Materials

The following supporting information can be downloaded at: https://www.mdpi.com/article/10.3390/genes15081098/s1, Figure S1: *IGFBP2* expression is increased in Huh7 cells transfected with FOG2^S657G^; Table S1: Top Differentially expressed genes in iHeps.

## References

[1] Shah P.A., Patil R., Harrison S.A. (2023). NAFLD-related hepatocellular carcinoma: The growing challenge. Hepatology.

[2] Zhou B., Ma Q., Kong S.W., Hu Y., Campbell P.H., McGowan F.X., Ackerman K.G., Wu B., Zhou B., Tevosian S.G. (2009). Fog2 is critical for cardiac function and maintenance of coronary vasculature in the adult mouse heart. J. Clin. Investig..

[3] Crispino J.D., Lodish M.B., Thurberg B.L., Litovsky S.H., Collins T., Molkentin J.D., Orkin S.H. (2001). Proper coronary vascular development and heart morphogenesis depend on interaction of GATA-4 with FOG cofactors. Genes. Dev..

[4] Guerraty M.A., Verma S., Ko Y.A., McQuillan M.A., Conlon D., Tobias J.W., Levin M.G., Haury W., Zhang C., Judy R. (2023). FOG2 coding variant Ser657Gly is associated with Coronary Microvascular Disease through altered hypoxia-mediated gene transcription. medRxiv.

[5] Guo Y., Yu J., Deng J., Liu B., Xiao Y., Li K., Xiao F., Yuan F., Liu Y., Chen S. (2016). A Novel Function of Hepatic FOG2 in Insulin Sensitivity and Lipid Metabolism Through PPARalpha. Diabetes.

[6] Xiao Y., Batmanov K., Hu W., Zhu K., Tom A.Y., Guan D., Jiang C., Cheng L., McCright S.J., Yang E.C. (2023). Hepatocytes demarcated by EphB2 contribute to the progression of nonalcoholic steatohepatitis. Sci. Transl. Med..

[7] Luo Y., Wang X., Ma L., Ma Z., Li S., Fang X., Ma X. (2020). Bioinformatics analyses and biological function of lncRNA ZFPM2-AS1 and ZFPM2 gene in hepatocellular carcinoma. Oncol. Lett..

[8] Karczewski K.J., Solomonson M., Chao K.R., Goodrich J.K., Tiao G., Lu W., Riley-Gillis B.M., Tsai E.A., Kim H.I., Zheng X. (2022). Systematic single-variant and gene-based association testing of thousands of phenotypes in 394,841 UK Biobank exomes. Cell Genom..

[9] Verma A., Damrauer S.M., Naseer N., Weaver J., Kripke C.M., Guare L., Sirugo G., Kember R.L., Drivas T.G., Dudek S.M. (2022). The Penn Medicine BioBank: Towards a Genomics-Enabled Learning Healthcare System to Accelerate Precision Medicine in a Diverse Population. J. Pers. Med..

[10] All of Us Research Program Genomics I. (2024). Genomic data in the All of Us Research Program. Nature.

[11] Verma A., Huffman J.E., Rodriguez A., Conery M., Liu M., Ho Y.L., Kim Y., Heise D.A., Guare L., Panickan V.A. (2024). Diversity and scale: Genetic architecture of 2068 traits in the VA Million Veteran Program. Science.

[12] Zhou W., Nielsen J.B., Fritsche L.G., Dey R., Gabrielsen M.E., Wolford B.N., LeFaive J., VandeHaar P., Gagliano S.A., Gifford A. (2018). Efficiently controlling for case-control imbalance and sample relatedness in large-scale genetic association studies. Nat. Genet..

[13] Ko Y.A., Sirugo G., Guerraty M.A., Gao Y., Raedschelders K., Kember R., Vujkovic M., Sheth S., Marchadier D., Munshi A.A. (2019). Proteomic Phenome-Wide Association Study With Penn Medicine Biobank Identifies Potential Novel Biomarkers for Disease Diagnosis in 400 African Americans. Circulation.

[14] Pashos E.E., Park Y., Wang X., Raghavan A., Yang W., Abbey D., Peters D.T., Arbelaez J., Hernandez M., Kuperwasser N. (2017). Large, Diverse Population Cohorts of hiPSCs and Derived Hepatocyte-like Cells Reveal Functional Genetic Variation at Blood Lipid-Associated Loci. Cell Stem Cell.

[15] Fahlbusch P., Knebel B., Hörbelt T., Barbosa D.M., Nikolic A., Jacob S., Al-Hasani H., Van De Velde F., Van Nieuwenhove Y., Müller-Wieland D. (2020). Physiological Disturbance in Fatty Liver Energy Metabolism Converges on IGFBP2 Abundance and Regulation in Mice and Men. Int. J. Mol. Sci..

[16] Chen X., Tang Y., Chen S., Ling W., Wang Q. (2021). IGFBP-2 as a biomarker in NAFLD improves hepatic steatosis: An integrated bioinformatics and experimental study. Endocr. Connect..

[17] Panwar V., Singh A., Bhatt M., Tonk R.K., Azizov S., Raza A.S., Sengupta S., Kumar D., Garg M. (2023). Multifaceted role of mTOR (mammalian target of rapamycin) signaling pathway in human health and disease. Signal Transduct. Target. Ther..

[18] Ricoult S.J., Manning B.D. (2013). The multifaceted role of mTORC1 in the control of lipid metabolism. EMBO Rep..

[19] Goupille O., Kadri Z., Langele A., Luccantoni S., Badoual C., Leboulch P., Chretien S. (2019). The integrity of the FOG-2 LXCXE pRb-binding motif is required for small intestine homeostasis. Exp. Physiol..

[20] Goupille O., Penglong T., Kadri Z., Granger-Locatelli M., Denis R., Luquet S., Badoual C., Fucharoen S., Maouche-Chretien L., Leboulch P. (2017). The LXCXE Retinoblastoma Protein-Binding Motif of FOG-2 Regulates Adipogenesis. Cell Rep..

[21] Hyun S., Lee J.H., Jin H., Nam J., Namkoong B., Lee G., Chung J., Kim V.N. (2009). Conserved MicroRNA miR-8/miR-200 and its target USH/FOG2 control growth by regulating PI3K. Cell.

[22] Pizzuti A., Sarkozy A., Newton A.L., Conti E., Flex E., Cristina Digilio M., Amati F., Gianni D., Tandoi C., Marino B. (2003). Mutations ofZFPM2/FOG2 gene in sporadic cases of tetralogy of Fallot. Human. Mutation.

[23] Fairfield C.J., Drake T.M., Pius R., Bretherick A.D., Campbell A., Clark D.W., Fallowfield J.A., Hayward C., Henderson N.C., Joshi P.K. (2022). Genome-Wide Association Study of NAFLD Using Electronic Health Records. Hepatol. Commun..

[24] Miao Z., Garske K.M., Pan D.Z., Koka A., Kaminska D., Mannisto V., Sinsheimer J.S., Pihlajamaki J., Pajukanta P. (2022). Identification of 90 NAFLD GWAS loci and establishment of NAFLD PRS and causal role of NAFLD in coronary artery disease. HGG Adv..

[25] Pan J.J., Fallon M.B. (2014). Gender and racial differences in nonalcoholic fatty liver disease. World J. Hepatol..

[26] Kalia H.S., Gaglio P.J. (2016). The Prevalence and Pathobiology of Nonalcoholic Fatty Liver Disease in Patients of Different Races or Ethnicities. Clin. Liver Dis..

[27] Sherif Z.A., Saeed A., Ghavimi S., Nouraie S.M., Laiyemo A.O., Brim H., Ashktorab H. (2016). Global Epidemiology of Nonalcoholic Fatty Liver Disease and Perspectives on US Minority Populations. Dig. Dis. Sci..

[28] Fry A., Littlejohns T.J., Sudlow C., Doherty N., Adamska L., Sprosen T., Collins R., Allen N.E. (2017). Comparison of Sociodemographic and Health-Related Characteristics of UK Biobank Participants With Those of the General Population. Am. J. Epidemiol..

[29] Vujkovic M., Ramdas S., Lorenz K.M., Guo X., Darlay R., Cordell H.J., He J., Gindin Y., Chung C., Myers R.P. (2022). A multiancestry genome-wide association study of unexplained chronic ALT elevation as a proxy for nonalcoholic fatty liver disease with histological and radiological validation. Nat. Genet..

[30] Sveinbjornsson G., Ulfarsson M.O., Thorolfsdottir R.B., Jonsson B.A., Einarsson E., Gunnlaugsson G., Rognvaldsson S., Arnar D.O., Baldvinsson M., Bjarnason R.G. (2022). Multiomics study of nonalcoholic fatty liver disease. Nat. Genet..

[31] Lin Y.H., Wei Y., Zeng Q., Wang Y., Pagani C.A., Li L., Zhu M., Wang Z., Hsieh M.H., Corbitt N. (2023). IGFBP2 expressing midlobular hepatocytes preferentially contribute to liver homeostasis and regeneration. Cell Stem Cell.

[32] Powell E.E., Wong V.W., Rinella M. (2021). Non-alcoholic fatty liver disease. Lancet.

[33] Balduzzi S., Rucker G., Schwarzer G. (2019). How to perform a meta-analysis with R: A practical tutorial. Evid. Based Ment. Health.

[34] Folch J., Lees M., Sloane Stanley G.H. (1957). A simple method for the isolation and purification of total lipides from animal tissues. J. Biol. Chem..

