# Supplementary material for: Genotype-First Approach Identifies an Association between rs28374544/FOG2S657G and Liver Disease through Alterations in mTORC1 Signaling
Source: Genes (Basel). 2024 Aug 21;15(8):1098. doi: 10.3390/genes15081098 (PMC11353451; doi:10.3390/genes15081098)
Supplement: Supplementary file 1 [file genes-15-01098-s001.zip › genes-3146150-supplementary.pdf]

Supplemental Table S1- Top Differentially expressed genes in iHeps

Feature ID	P-value (AG vs AA)	FDR step up (AG vs AA)	Ratio (AG vs AA)	Fold change (AG vs AA)	LSMean (AG)	LSMean (AA)
IGFBP2	2.10E-04	0.95	1.38	1.38	1526.54	1104.37
GPI	5.76E-04	0.95	1.13	1.13	291.52	257.50
SUN1	6.73E-04	0.95	1.18	1.18	102.38	86.53
SLC2A1	9.44E-04	1.00	1.42	1.42	432.11	305.09
ENO1	1.17E-03	1.00	1.11	1.11	3041.28	2739.20
DCAF13	1.20E-03	1.00	0.88	-1.13	149.79	169.74
ALDOA	1.56E-03	1.00	1.15	1.15	1863.65	1617.92
PKM	1.69E-03	1.00	1.17	1.17	1382.33	1177.81
KDM2B	2.90E-03	1.00	1.15	1.15	64.98	56.28
RABGGTB	2.93E-03	1.00	0.87	-1.14	104.27	119.26
RPL36AL	2.94E-03	1.00	0.86	-1.17	252.42	294.15
MRPL36	2.99E-03	1.00	0.85	-1.17	58.76	68.83
TARDBP	3.49E-03	1.00	0.90	-1.11	199.13	220.69
PGK1	3.52E-03	1.00	1.24	1.24	2513.60	2020.83
UCHL1	3.78E-03	1.00	0.90	-1.11	711.11	788.75
SEPTIN9	3.81E-03	1.00	1.14	1.14	215.29	188.08
PPT1	4.32E-03	1.00	0.89	-1.12	133.44	149.90
CD9	5.60E-03	1.00	1.16	1.16	274.66	237.74
EEF1E1	5.66E-03	1.00	0.88	-1.13	120.86	136.90
NOP16	6.82E-03	1.00	0.82	-1.21	45.41	55.11

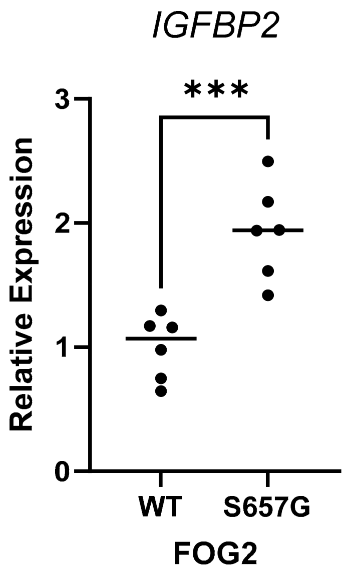

Figure S1- ***IGFBP2*** **expression is increased in Huh7 cells transfected with FOG2^{S657G}.** Huh7 cells were transiently transfected with vectors expressing FOG2 (WT), or FOG2^{S657G}(S657G). Gene expression measured 72 hours post-transfection, using qRT-PCR showed increased expression of IGFBP2 with S657G expression.

Regeneron Genetics Center Banner Author List and Contribution Statements

RGC Management and Leadership Team

Goncalo Abecasis, PhD , Aris Baras, M.D. , Michael Cantor, M.D. , Giovanni Coppola, M.D. , Andrew Deubler , Aris Economides, Ph.D. , Luca A. Lotta, M.D., Ph.D. , John D. Overton, Ph.D. , Jeffrey G. Reid, Ph.D. , Katherine Siminovitch, M.D. , Alan Shuldiner, M.D.

Sequencing and Lab Operations

Christina Beechert , Caitlin Forsythe, M.S. , Erin D. Fuller , Zhenhua Gu, M.S. , Michael Lattari , Alexander Lopez, M.S. , John D. Overton, Ph.D. , Maria Sotiropoulos Padilla, M.S. , Manasi Pradhan, M.S. , Kia Manoochehri, B.S. , Thomas D. Schleicher, M.S. , Louis Widom , Sarah E. Wolf, M.S. , Ricardo H. Ulloa, B.S.

Clinical Informatics

Amelia Averitt, Ph.D. , Nilanjana Banerjee, Ph.D. , Michael Cantor, M.D. , Dadong Li, Ph.D. , Sameer Malhotra, M.D. , Deepika Sharma, MHI , Jeffrey Staples , Ph.D.

Genome Informatics

Xiaodong Bai, Ph.D. , Suganthi Balasubramanian, Ph.D. , Suying Bao, Ph.D. , Boris Boutkov, Ph.D. , Siying Chen, Ph.D. , Gisu Eom, B.S. , Lukas Habegger, Ph.D. , Alicia Hawes, B.S. , Shareef Khalid , Olga Krasheninina, M.S. , Rouel Lanche, B.S. , Adam J. Mansfield, B.A. , Evan K. Maxwell, Ph.D. , George Mitra, B.A. , Mona Nafde, M.S. , Sean O'Keeffe, Ph.D. , Max Orelus, B.B.A. , Razvan Panea, Ph.D. , Tommy Polanco, B.A. , Ayesha Rasool, M.S. , Jeffrey G. Reid, Ph.D. , William Salerno, Ph.D. , Jeffrey C. Staples, Ph.D. , Kathie Sun, Ph.D.

Analytical Genomics and Data Science

Goncalo Abecasis, D.Phil. , Joshua Backman, Ph.D. , Amy Damask, Ph.D. , Lee Dobbyn, Ph.D. , Manuel Allen Revez Ferreira, Ph.D. , Arkopravo Ghosh, M.S. , Christopher Gillies, Ph.D. , Lauren Gurski, B.S. , Eric Jorgenson, Ph.D. , Hyun Min Kang, Ph.D. , Michael Kessler, Ph.D. , Jack Kosmicki, Ph.D. , Alexander Li , Ph.D. , Nan Lin, Ph.D. , Daren Liu, M.S. , Adam Locke, Ph.D. , Jonathan Marchini, Ph.D. , Anthony Marcketta, M.S. , Joelle Mbatchou, Ph.D. , Arden Moscati, Ph.D. , Charles Paulding, Ph.D. , Carlo Sidore, Ph.D. , Eli Stahl, Ph.D. , Kyoko Watanabe, Ph.D. , Bin Ye, Ph.D. , Blair Zhang, Ph.D. , Andrey Ziyatdinov, Ph.D.

Therapeutic Area Genetics

Ariane Ayer, B.S. , Aysegul Guvenek, Ph.D. , George Hindy, Ph.D. , Giovanni Coppola, M.D. , Jan Freudenberg, M.D. , Jonas Bovijn M.D. , Katherine Siminovitch, M.D. , Kavita Praveen, Ph.D. , Luca A. Lotta, M.D. , Manav Kapoor, Ph.D. , Mary Haas, Ph.D. , Moeen Riaz , Ph.D. , Niek Verweij, Ph.D. , Olukayode Sosina, Ph.D. , Parsa Akbari, Ph.D. , Priyanka

Nakka, Ph.D. , Sahar Gelfman, Ph.D. , Sujit Gokhale, B.E. , Tanima De, Ph.D. , Veera Rajagopal, Ph.D. , Alan Shuldiner, M.D. , Bin Ye, Ph.D. , Gannie Tzoneva, Ph.D. , Juan Rodriguez-Flores, Ph.D.

Research Program Management & Strategic Initiatives

Esteban Chen, M.S. , Marcus B. Jones, Ph.D. , Michelle G. LeBlanc, Ph.D. , Jason Mighty, Ph.D. , Lyndon J. Mitnaul, Ph.D. , Nirupama Nishtala, Ph.D. , Nadia Rana, Ph.D. , Jaimee Hernandez
